# The Role of Subthreshold Micropulse Yellow Laser as an Alternative Option for the Treatment of Refractory Postoperative Cystoid Macular Edema

**DOI:** 10.3390/jcm9041066

**Published:** 2020-04-09

**Authors:** Tommaso Verdina, Rossella D’Aloisio, Andrea Lazzerini, Cecilia Ferrari, Edoardo Valerio, Rodolfo Mastropasqua, Gian Maria Cavallini

**Affiliations:** 1 Institute of Ophthalmology–University of Modena and Reggio Emilia, 41121 Modena, Italy; 2 Ophthalmology Clinic, Department of Medicine and Science of Ageing, University G. d’Annunzio Chieti-Pescara, 66100 Chieti, Italy; ross.daloisio@gmail.com

**Keywords:** post-surgical cystoid macular edema, micropulse yellow laser, cataract surgery, refractory macular edema

## Abstract

Background: To evaluate the efficacy and the safety of subthreshold micropulse yellow laser (SMYL) in the treatment of chronic postoperative cystoid macular edema (PCME), which is refractory to standard therapies. Methods: A retrospective chart review of ten eyes of ten patients affected by refractory PCME who underwent SMYL was performed. Five PCME cases were subsequent to uncomplicated cataract surgery (CS), two cases to complicated CS (CCS) with posterior capsule rupture and three cases occurred after retinal detachment surgery (RD). All conditions were refractory to conventional treatments prior to SMYL interventions for at least 4 months, including nonsteroidal anti-inflammatory eyedrops, topical steroids, oral indomethacin, sub-Tenon’s triamcinolone injections and Dexamethasone intravitreal implants. All patients underwent one or more treatments with 577 nm SMYL photo-stimulation, with 7 × 7 grids with confluent spots and a 5% duty cycle covering the whole edematous retina, including the foveal center. Best corrected visual acuity (BCVA) and central macular thickness (CMT) were obtained using OCT, and evaluated before and after the treatment at 1, 2, 3 and 6-month follow-ups. Results: A complete subfoveal macular edema resolution was observed in all of the eyes, with statistically significant improvements in terms of BCVA and CMT in all of the follow-up timelines (at 6 months, *p* = 0.002 and *p* = 0.005, respectively). The mean number of laser treatments was 1.3. At the final follow-up, a complete subfoveal edema reabsorption was observed in all patients with visual acuity improvement. No complications were observed in any case. Conclusions: SMYL seems to be a safe and effective treatment for the long-term resolution of refractory PCME and may be a useful alternative to expensive and invasive therapeutic options.

## 1. Introduction

Postoperative cystoid macular edema (PCME) is defined as the presence of intraretinal cystoid spaces subsequent to ocular surgery, or central subfield macular thickening in optical coherence tomography (OCT) examination [1]. PCME occurring after cataract surgery, also known as Irvine–Gass syndrome, was firstly described as a new disease entity by Irvine in 1953 [2] and subsequently studied with fluorescein angiography by Gass and Norton [3], and is still one of the most common causes of visual loss after cataract surgery [4,5]. The incidence of PCME (1–2% of clinically significant PCME in patients with no risk factors) has increased significantly with the advent of OCT, as well as the early assessment of other chronic macular pathologies [6].

PCME can also occur after vitreoretinal surgery, with an estimated incidence at structural OCT of up to 47% after an ERM peel [7] and 16% after a retinal detachment repair [8].

It is well established that postoperative inflammation, due to surgical manipulation associated to the disruption of the blood–retinal barrier, is one of the major causes of PCME [9]. In most cases, PCME resolves without the need for therapy; however, it sometimes requires treatment. Over the years, many therapy options have been proposed, such as anti-inflammatory eye drops, corticosteroids and anti-vascular endothelial growth factor (anti-VEGF) injections [1,10,11].

Some cases which are refractory to standard therapies are encountered in which a complete fluid resolution can be difficult, leading to long-term visual deterioration.

The use of subthreshold micropulse yellow laser (SMYL) has widespread benefits for the treatment of different macular disorders [12]. Compared to a conventional continuous wavelength laser, the micropulse yellow laser is a revolutionary alternative, as tissue is preserved due to its use of a photo-stimulation process with repetitive short pulses at low temperatures. Yellow light has an excellent absorption rate for O2 Hb, and is not absorbed by foveal pigments such as Lutein and Zeaxantin, thus allowing central macular edema treatment without foveal damage.

The efficacy of the SMYL for some macular disorders, such as diabetic macular edema (DME), retinal vein occlusion (RVO) and central serous chorioretinopathy (CSC), has been described well in the literature and was also reviewed by Gawecki M. [12]; however, there are no cases reporting on its use in the treatment of PCME.

We report a series of cases of chronic refractory PCME caused by different surgical procedures (uncomplicated cataract surgery, complicated cataract surgery and vitreoretinal surgery) that were completely resolved with the SMYL treatment.

## 2. Experimental Section

### 2.1. Study Participants

A retrospective review of the ophthalmological charts of 10 consecutive patients affected by chronic PCME, who were treated with a subthreshold micropulse yellow laser (SMYL) at the Institute of Ophthalmology, University of Modena (Italy), was performed. The patient treatments were registered between October 2018 and May 2019. The study adhered to the tenets of the Declaration of Helsinki, written informed consent was obtained from each patient and our Institutional Review Board approved the retrospective consecutive chart review.

The inclusion criteria were as follows: (1) chronic PCME which was refractory to conventional treatments for at least 4 months, including non-steroidal anti-inflammatory eyedrops and tablets (oral indomethacin), topic steroids, sub-Tenon’s triamcinolone injections and Dexamethasone intravitreal implants; (2) age ≥ 18 years old, with no previous treatments with SMYL. Exclusion criteria were other chorioretinal diseases including active AMD, glaucoma, optic nerve disease/abnormalities and diabetic retinopathy.

We divided patients into three sub-groups depending on the surgery (UC, CC and RD) (C = cataract surgery, CC = complicated cataract surgery, RD = retinal detachment).

### 2.2. Study Evaluations

Best corrected visual acuity (BCVA) was evaluated with the Early Treatment Diabetic Retinopathy Study (ETDRS) charts at 4 months and the assessment was in Snellen. We converted the results into LogMAR values.

Central macular thickness (CMT) was assessed using spectral domain OCT (OCT-SLO, Optos, Scotland, UK). The OCT acquisition protocol consisted of a macular cube 512 × 128 scan pattern in which a 6.0 × 6.0 mm region of the retina was scanned (for a total of 65,536 sampled points) and 5 horizontal raster lines which passed through the fovea. The macular thickness value was obtained using the integrated OCT software. Macular edema was defined as the presence of intraretinal and subfoveal hyporeflective spaces. Moreover, the OCT examination during the follow-up included the evaluation of the integrity of the ellipsoid zone (EZ), which refers to the presence of photoreceptors.

Post-operative values were recorded at 1, 2, 3 and 6 months after laser treatment, in terms of BCVA and CMT.

### 2.3. Laser Treatment

The persistent macular edema was treated in all cases with 577 nm SMYL photo-stimulation (IRIDEX IQ 577™, IRIDEX, Mountain View, CA, USA) using a 1.06 × laser magnification lens (Goldmann’s three-mirror fundus lens, Volk Optical Inc., Mentor, OH USA). Initially, a continuous wave test was performed to determine the correct minimum threshold power. In a non-edematous area in the vascular arcades, at more than 3 disc diameters from the foveal center, a 200 µm diameter spot was tested with pulse duration of 200 msec and power of 50 mW. The power was augmented at 10 mW increments (whilst advancing the laser to non-edematous areas immediately beside the previous test site) until a barely-visible tissue reaction (white color) was observed.

The micropulse laser therapy was then performed on the edema site, switching to a 5% duty cycle and adjusting the power to four times the test spot threshold with 200 ms exposure, using 4 grids (7 × 7) with confluent spots of 200 µm (0.00 spacing) covering the whole edematous area, including the foveal center. The setting, including spot size, lens and duration, remained the same as it was in the test spot.

Bromfenac 0.09% eye drops twice/daily for one month were prescribed post-intervention in all cases.

### 2.4. Statistical Analysis

The study data were collected and divided into pre- and post-treatment values for each patient and compared. For statistical analysis, we used an Excel database (Microsoft Excel 2010, Microsoft Office Professional Plus 2010) and Stata 13.1 software (StataCorp LP, College Station, TX, USA) was employed for Student’s *t*-test and the Wilcoxon rank sum test. Given the non-normal distribution verified by the Shapiro–Wilk test and the small sample size, the pre- and post-rehabilitation values were compared with the one-sided Wilcoxon Signed Rank Test. Since the number of comparisons was small, the *p*-values were not corrected by the number of comparisons made in the Wilcoxon test run. The significance is established for values of *p* < 0.05.

## 3. Results

Ten eyes of ten patients (four males and six females, five right eyes and five left eyes) were included in the study. The average age was 72 ± 15 years old (range 36–89). Their demographic characteristics and PCME information, including previous treatments, are shown in Table 1.

Five PCME cases were subsequent to UC, two cases to CC with posterior capsule rupture and three cases occurred after RD.

All of the patients were previously treated with topical non-steroidal anti-inflammatory drugs (NSAIDs)(Nepafenac 3 mg once/day or Bromfenac 0.09% twice/day). Other treatments, including oral NSAIDs (50 mg of Indomethacin twice/day), Triamcinolone sub-Tenon’s injections (TA), Dexametazone intravitreal implants (Ozurdex) and anti-VEGF intravitreal injections, are described in Table 1. In one case, three injections of antiVEGF (Lucentis) were previously performed, as it was thought to be a DME.

**Figure 1:**
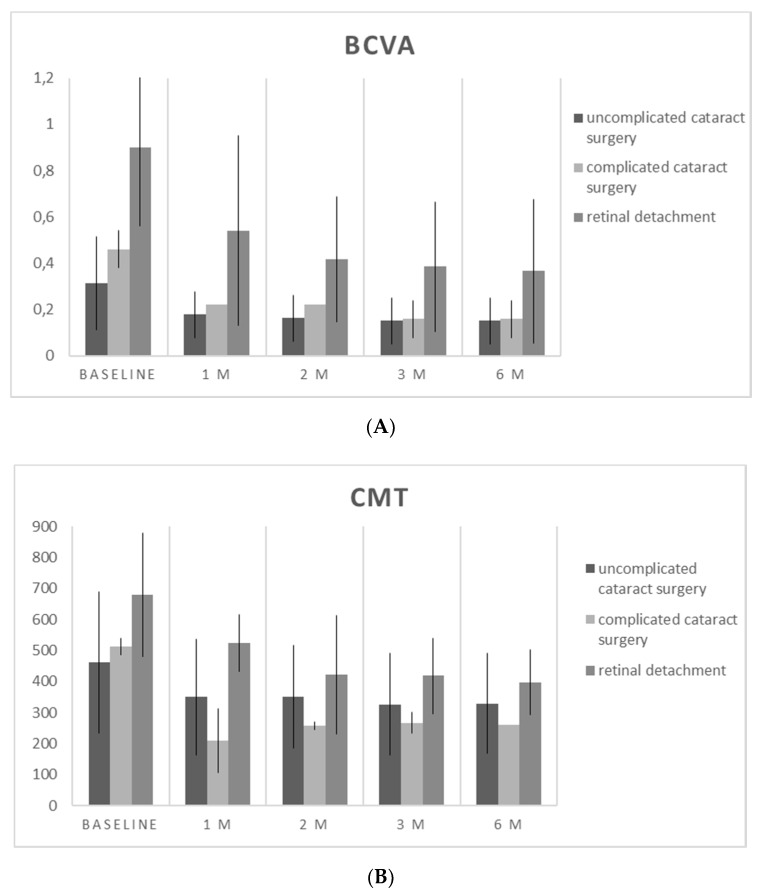
(**A**) Best corrected visual acuity (BCVA) variation during the follow-up for the three different sub-groups. Values are presented in logMar. (**B**) Central macular thickness (CMT) variation during the follow-up for the three different sub-groups. Values are presented in microns.

**Figure 2:**
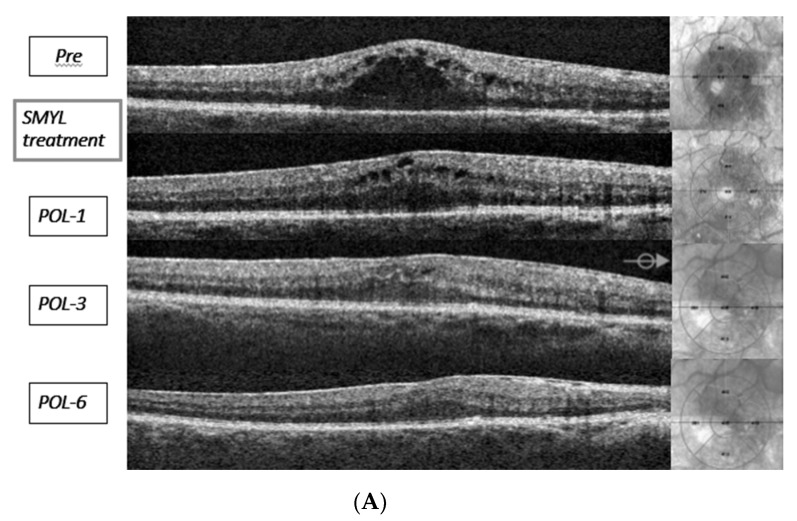

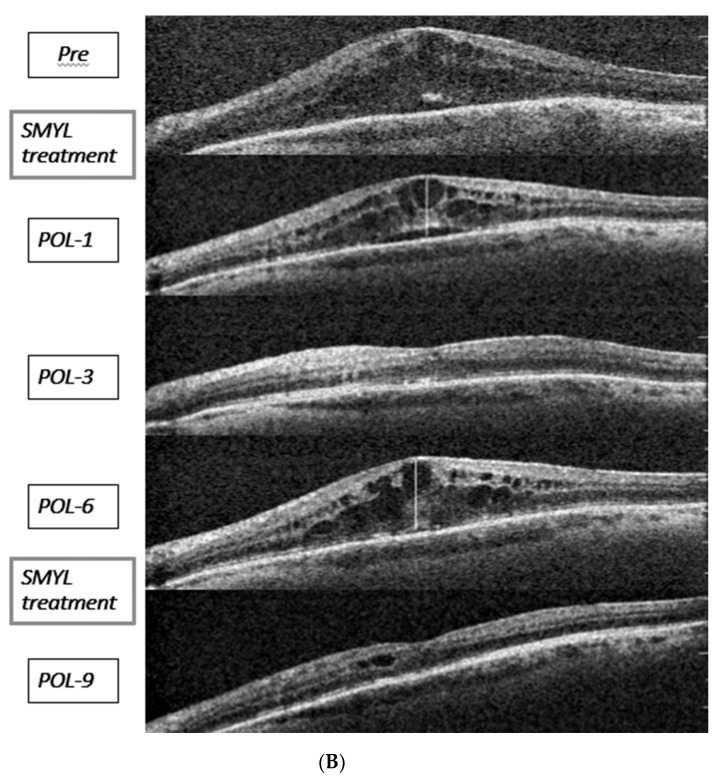
(**A**) OCT images of macular edema resolution at the 6-month follow-up in patient n.1 (POL-1 = post-operative laser month one), (**B**) OCT images of macular edema resolution after two SMYL treatments in patient n.8.

BCVA and CMT improved significantly in all follow-up time points. All results are summarized in Figure 1 and Figure 2, and Table 2.

Considering the three different sub-groups (UC, CC and RD), we noticed that BCVA and CMT improved in all groups (Figure 1), with significant improvements for BCVA in the RD group (*p* = 0.016 at 6 months) and for CMT in the UC group (*p* = 0.031 at 6 months).

At OCT, an EZ band was present in all cases in the 6-month follow-up, with the exception of P8, who presented a disruption of the EZ in the foveal area at the end of the follow-up.

In all patients, PCME was resolved with one single SMYL treatment, except in two cases.

In case n.6 (P6), three treatments were needed: two subfoveal edema relapses were observed at three months from the initial laser intervention and four months from the second laser intervention (Figure 2A). In both cases, the edema was successfully treated. The final patient follow-up was performed at 4 months from the third laser treatment, and no edema was evident.

In case n. 8, two treatments had been performed: one edema relapse was observed at the 5-month follow-up from the initial laser intervention. After the second treatment, the subfoveal edema was resolved with a 6-month follow-up (Figure 2B).

The laser power used was between 300 and 400 mW in all cases, depending on the parameters explained in the method section. For re-treatment, we added 20 mW to the previous treatment.

In P8, P9 and P10, SMYL was performed after silicone oil extraction.

## 4. Discussion

The efficacy of SMYL for the treatment of some retinal diseases, such as central serous chorioretinopathy (CSC), diabetic macular edema (DME) and macular edema secondary to retinal vein occlusion (RVO), has been previously reported in the literature [12]. However, to the best of our knowledge, there are no other works reporting on its application for chronic refractory PCME.

Irvine–Gass syndrome is still one of the main causes of visual impairment after cataract surgery and is considered a late postoperative complication [13].

Some of the risk factors for PCME development have been described, including diabetes mellitus, posterior capsule rupture during cataract surgery, and the previous diagnosis of epiretinal membranes, uveitis, retinal vein occlusion and retinal detachment [14].

In our cohort of patients, two cases of PCME were consequent to cataract surgery which was complicated by posterior capsule rupture, and three cases were after retinal detachment surgery.

Several treatments have been proposed, such as anti-inflammatory eye drops, dexamethasone intravitreal implants, anti-VEGF injections, argon laser photocoagulation and even vitreoretinal surgery [15].

In a retrospective chart review of 100 eyes with refractory retinal diseases, such as macular edema secondary to retinal vein occlusion (RVO), diabetic retinopathy (DME), posterior noninfectious uveitis (NIU), and pseudophakic Irvine–Gass syndrome (IGS), intravitreal dexamethasone implantation appeared to be well tolerated in all pathological conditions, with a consistent improvement of anatomical outcomes [16]. However, the positive anatomical outcomes of dexamethasone were often not correlated with a gain in terms of visual acuity, and patients who were treated earlier had better outcomes.

Furthermore, corticosteroid implants and repeated sub-Tenon’s triamcinolone or anti-VEGF intravitreal injections are all invasive therapies, due to their association with local complications such as rhegmatogenous retinal detachment, endophthalmitis, intraocular pressure elevation, ocular hemorrhage, and systemic complications including thromboembolic events [17].

Pars plana vitrectomy, which is considered an option for the treatment of PCME, has been associated with complications ranging from iatrogenic tears to choroidal hemorrhage [18].

Some studies have investigated the efficacy and safety of SMYL in treating different macular conditions [9,12]. Indeed, it induces the expression of anti-angiogenic and restorative biological factors within the retinal pigment epithelium cells, thus leading to the overexpression of both pigment epithelium derived factor (PEDF) and vascular endothelial growth factor (VEGF) inhibitors, and the sub-regulation of VEGF inducers and permeability factors [19,20]. Moreover, sublethal photothermal stimulation induces the expression of heat shock protein (HSP), thereby normalizing the response of the cytokines responsible for different retinal disorders and reducing chronic inflammation without any tissue damage [21]. Almeida et al. [22] have already observed a significantly decreased macular volume in patients with PCME receiving anti-inflammatory eyedrops, suggesting the main role of VEGF and other cytokines of inflammation in PCME development. The impairment of choroidal circulation and the dysfunction of the RPE barrier are also believed to play an important role in the pathophysiology of the disease [23].

Our case series showed the excellent efficacy of SMYL for the treatment of PCME, with both significant anatomical and functional recovery at the 6-month follow-up. In total, a 100% complete subfoveal edema resolution was found in our sample in all three different subgroups, without statistically significant differences between them. In our sample, two cases of subfoveal edema relapses were observed at three months from the initial laser intervention and four months from the second laser intervention in a single case of PCME consequent to complicated cataract surgery, which is known to increase the level of inflammation in comparison with uneventful cataract surgery. We also registered a case of subfoveal edema relapse at 6 months from the first treatment in a case of PCME consequent to RD surgery.

SMYL has demonstrated advantages in treating foveal region targeting RPE by establishing photo-stimulation, without negative thermal effects on the neural retina and deeper structures [9]. Furthermore, it is able to re-treat poor responses or relapses with anatomical and functional success, similarly to our recurrence case, which, after multiple laser sessions, showed complete subfoveal fluid reabsorption without RPE damages. Of note, in our cohort, the EZ was present in all cases at the 6-month follow-up; only in one case did the EZ appear slightly interrupted, likely due to long-standing edema (13 months) and the presence of RD involving the foveal area.

Referring to RD cases, it is known that vitrectomy may contribute to changes in intraocular pharmacokinetics; thus, micropulse lasers might work differently in vitrectomized eyes. Indeed, some previous animal and human studies reported that vitrectomy seems to increase vitreous oxygenation in the context of retinal ischemia [24,25,26]. Holekamp et al. detected a significantly higher mid-vitreous pO2 in eyes that had undergone previous vitreoretinal surgeries, compared with a healthy control group [27]. In addition, subthreshold micropulse lasers, whose pathophysiology is not fully understood, may induce the upregulation and downregulation of different vascular and permeability factors [28], probably enhancing the effect of the vitrectomy described above. However, in our study, we found statistically significant improvements in both anatomical (CMT) and functional (BCVA) parameters after micropulse laser treatment in all three groups without any significant differences between them, likely due to the small sample size and the lower baseline visual acuity of the RD group in comparison with the other two groups.

Our study has some limitations, such as the retrospective nature of the study, and the small sample of patients with PCME who were previously treated with different therapy options and who underwent SMYL at different time points after surgery. Furthermore, different types of surgery (uncomplicated cataract surgery, complicated cataract surgery, vitreoretinal surgery for retinal detachment) were considered in the analysis; however, a sub-analysis was performed in order to better investigate the efficacy of SMYL, and possible differences of PCME development and responsiveness to micropulse lasers depending on different surgeries. Finally, an FA or OCT-A would have been needed to exclude the presence of an underlying choroidal neovascularization; nevertheless, FA was not performed on any patients prior to the SMYL.

In conclusion, SMYL seems to be a safe and effective treatment for the short-term and relatively long-term (6 months) resolution of refractory PCME, and represents a useful alternative to expensive and invasive therapies, such as vitreoretinal surgery or repeated sub-Tenon’s triamcinolone or intravitreal injections, without any complications.

Undoubtedly, further studies with a wider sample and a longer follow-up are required to strengthen our results, and to investigate potential structural biomarkers of responsiveness; in particular, prospective comparative trials will be needed to investigate SYML as a first line of treatment in naïve cases of PCME in which the pathophysiology remains unclear.

## Figures and Tables

**Table 1:** Demographic data with surgical intervention and SMYL treatment.

ID	Age	Eye	PCME	Surgical Intervention	Duration PCME (Months)	Treatments Prior to SMYL	Baseline BCVA (LogMAR)	Baseline CMT (µm)	*n* of SMYL
P1	86	OD	UC	Phaco/IOL	4	NSAIDs	0.40	644	1
P2	80	OD	UC	Phaco/IOL	11	3 TA, 1 Ozurdex	0.40	303	1
P3	89	OD	UC	Phaco/IOL	15	NSAIDs	0.15	301	1
P4	74	OS	UC	Phaco/IOL	8	2 TA	0.15	290	1
P5	79	OS	UC	Phaco/IOL	10	3 Lucentis	0.52	770	1
P6	79	OD	CC	Phaco/IOL and PC-R +Scleral fixation same IOL	16	NSAIDs, 3 TA	0.52	532	3
P7	67	OS	CC	Phaco/IOL and PC-R + IC-IOL posterior	9	NSAIDs 2 TA	0.40	493	1
P8	73	OS	RD (m-Off)	PPV + SO	13	1 TA, 1 Ozurdex	1.30	600	2
P9	36	OD	RD (m-On)	PPV + SO	10	NSAIDs	0.70	890	1
P10	53	OD	RD (m-On)	PPV + SO	5	NSAIDs	0.70	550	1

BCVA= best corrected visual acuity, CMT= central macular thickness, UC = cataract surgery, CC = complicated cataract, RD = retinal detachment; m-Off = macula-off; m-On = macula-on PC-R = posterior capsule rupture; PPV = pars plana vitrectomy; SO = Silicon Oil; IC = Iris-claw; NSAIDs = oral no-steroidal anti-inflammatory drugs; TA = Triamcinolone sub-Tenon’s injection.

**Table 2:** Mean values of BCVA and CMT at baseline and post-treatment, with *p*-values.

	BCVA (LogMAR)	*p* Value (pre vs post)	CMT (µm)	*p* Value (pre vs post)
Baseline	0.52 ± 0.34		537.30 ± 202.18	
M1	0.30 ± 0.27	<0.001	374.60 ± 163.75	<0.001
M2	0.25 ± 0.18	<0.001	353.60 ± 125.30	<0.001
M3	0.22 ± 0.19	<0.001	342.40 ± 108.37	<0.001
M6	0.22 ± 0.19	<0.001	336.00 ± 100.30	<0.001

M = month.
